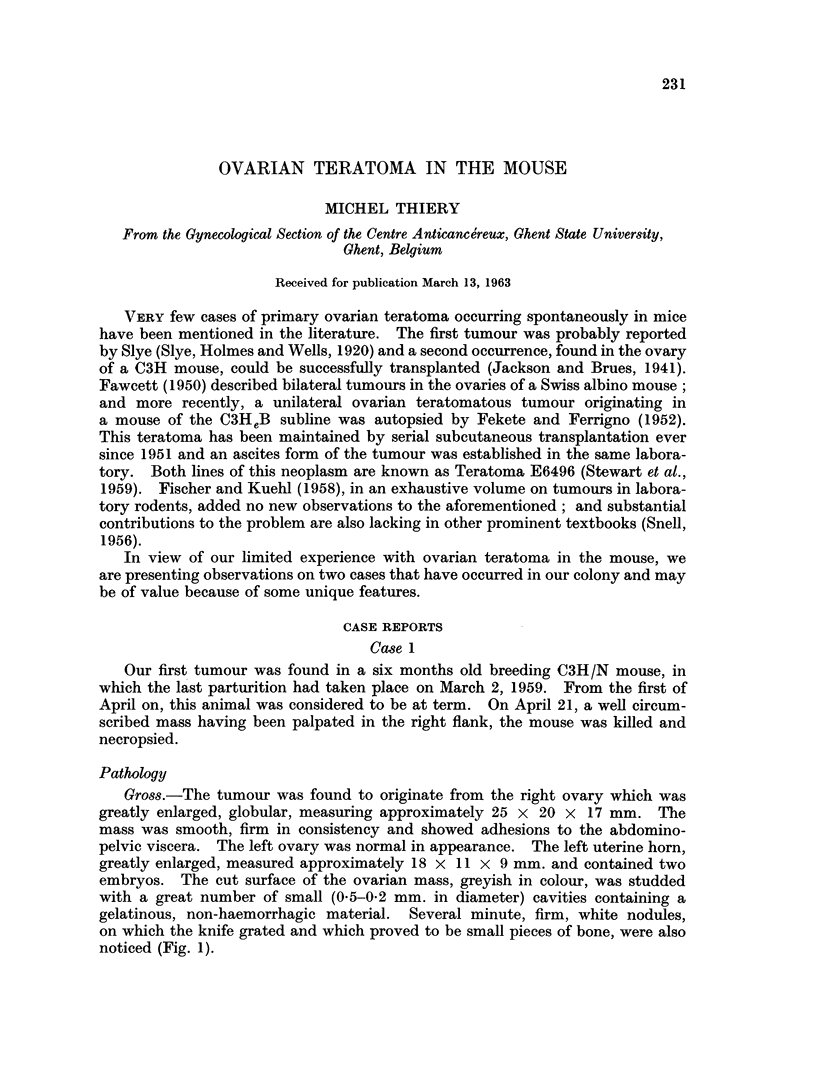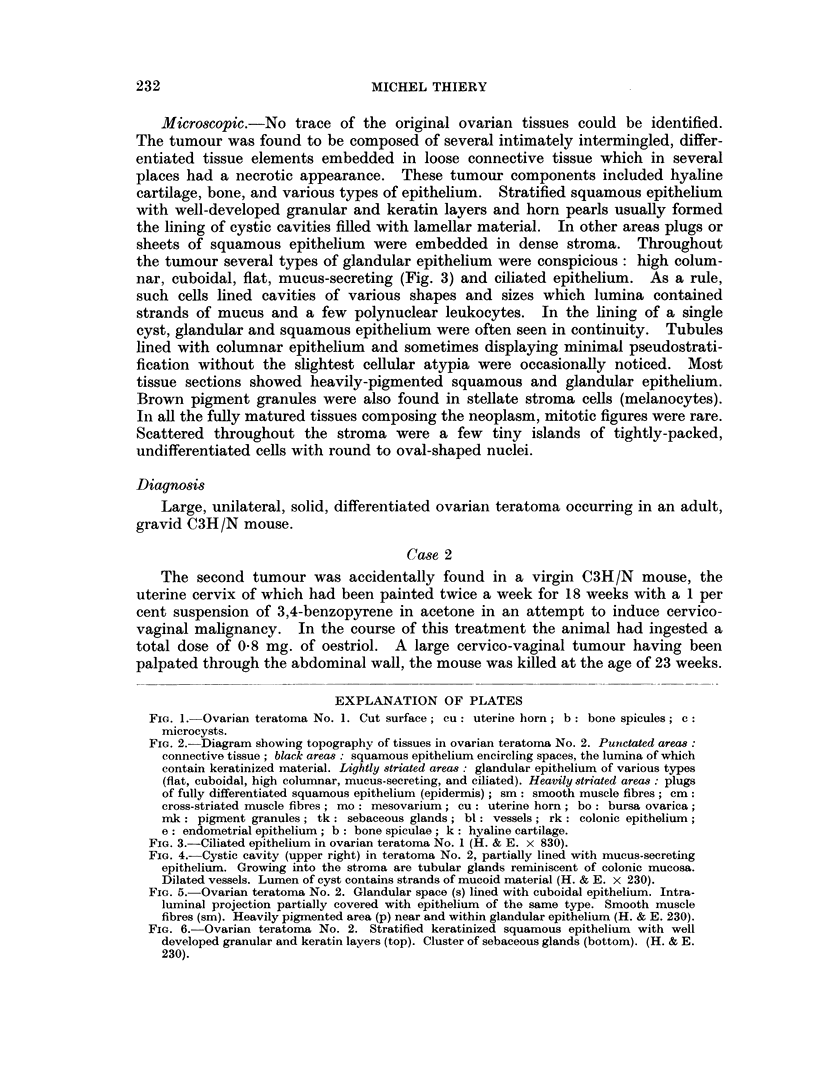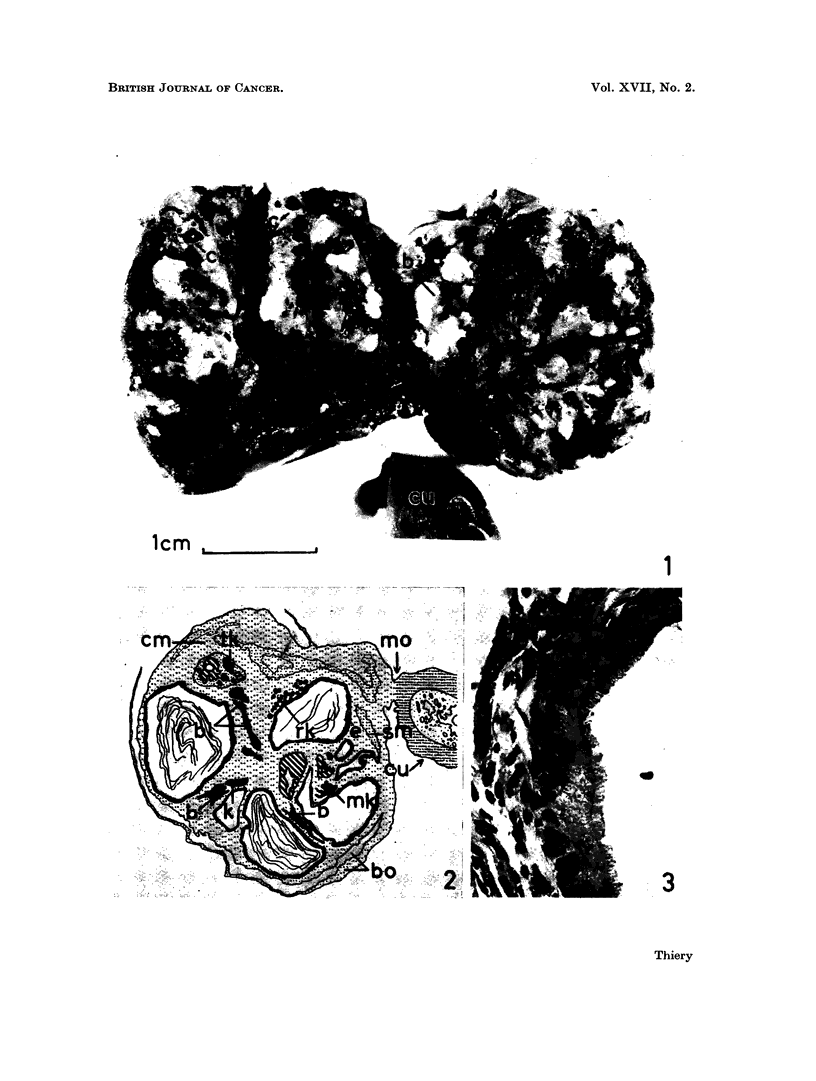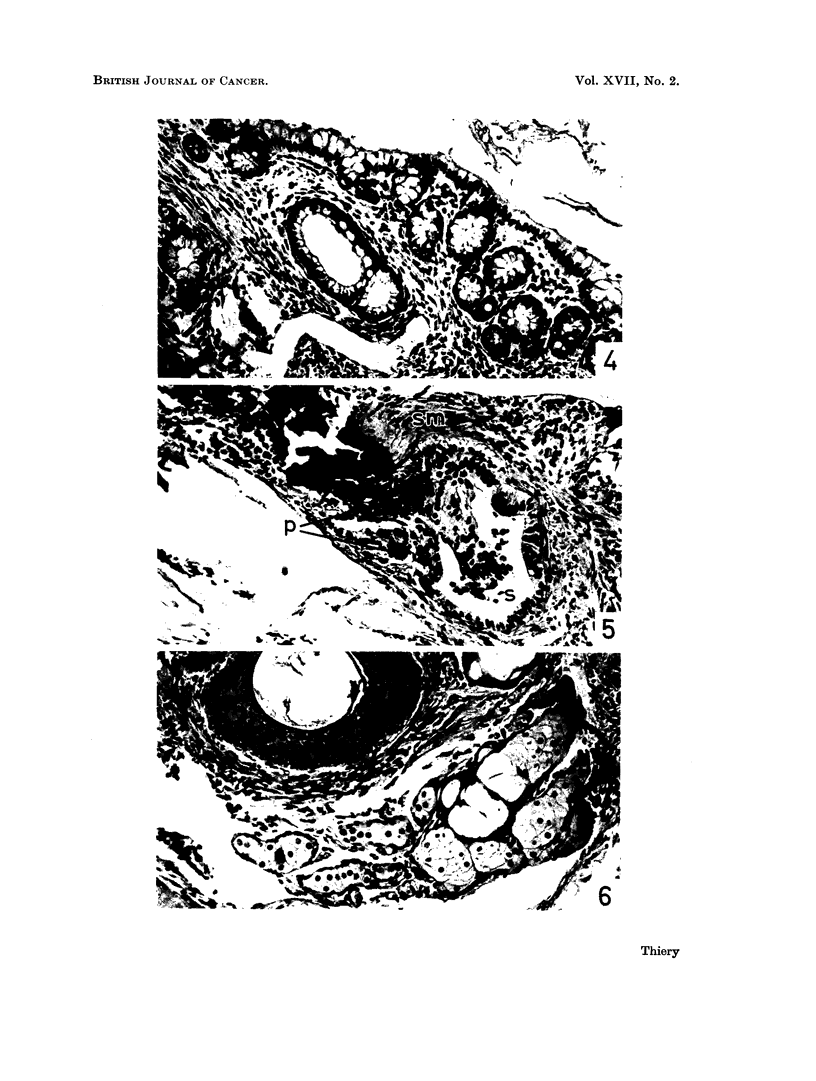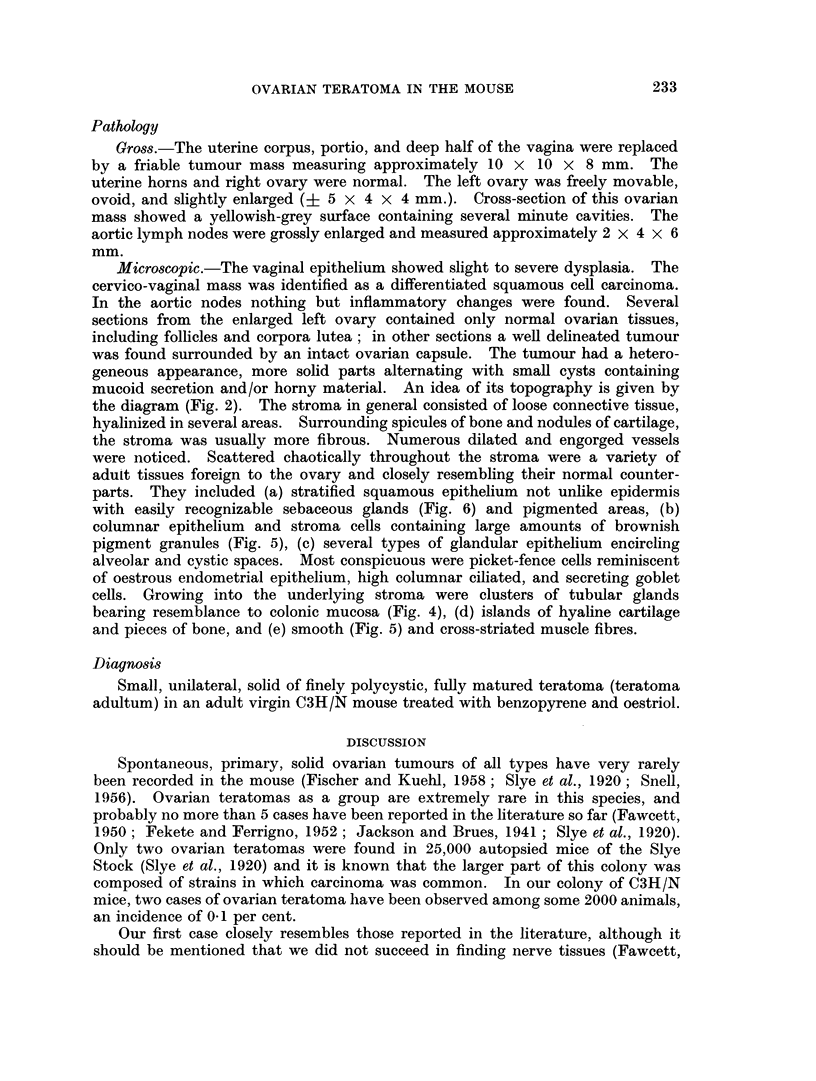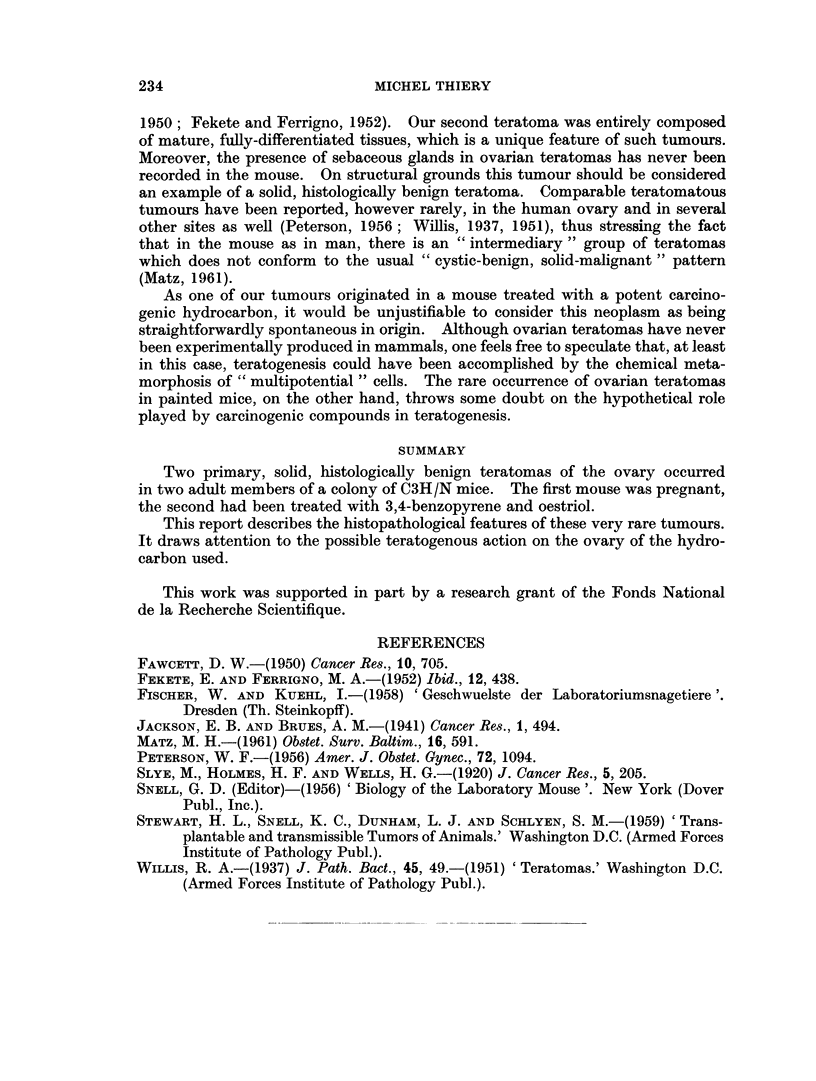# Ovarian Teratoma in the Mouse

**DOI:** 10.1038/bjc.1963.32

**Published:** 1963-06

**Authors:** Michel Thiery

## Abstract

**Images:**


### OVARIAN TERATOMA IN THE MOUSE

MICHEL THIERY

*From the Gynecological Section of the Centre Anticancéreux, Ghent State University, Ghent, Belgium*

Received for publication March 13, 1963

VERY few cases of primary ovarian teratoma occurring spontaneously in mice have been mentioned in the literature. The first tumour was probably reported by Slye (Slye, Holmes and Wells, 1920) and a second occurrence, found in the ovary of a C3H mouse, could be successfully transplanted (Jackson and Brues, 1941). Fawcett (1950) described bilateral tumours in the ovaries of a Swiss albino mouse ; and more recently, a unilateral ovarian teratomatous tumour originating in a mouse of the $C3H_eB$ subline was autopsied by Fekete and Ferrigno (1952). This teratoma has been maintained by serial subcutaneous transplantation ever since 1951 and an ascites form of the tumour was established in the same laboratory. Both lines of this neoplasm are known as Teratoma E6496 (Stewart *et al.*, 1959). Fischer and Kuehl (1958), in an exhaustive volume on tumours in laboratory rodents, added no new observations to the aforementioned ; and substantial contributions to the problem are also lacking in other prominent textbooks (Snell, 1956).

In view of our limited experience with ovarian teratoma in the mouse, we are presenting observations on two cases that have occurred in our colony and may be of value because of some unique features.

### CASE REPORTS

### *Case* 1

Our first tumour was found in a six months old breeding C3H/N mouse, in which the last parturition had taken place on March 2, 1959. From the first of April on, this animal was considered to be at term. On April 21, a well circumscribed mass having been palpated in the right flank, the mouse was killed and necropsied.

### *Pathology*

*Gross.*—The tumour was found to originate from the right ovary which was greatly enlarged, globular, measuring approximately 25 × 20 × 17 mm. The mass was smooth, firm in consistency and showed adhesions to the abdomino-pelvic viscera. The left ovary was normal in appearance. The left uterine horn, greatly enlarged, measured approximately 18 × 11 × 9 mm. and contained two embryos. The cut surface of the ovarian mass, greyish in colour, was studded with a great number of small (0·5–0·2 mm. in diameter) cavities containing a gelatinous, non-haemorrhagic material. Several minute, firm, white nodules, on which the knife grated and which proved to be small pieces of bone, were also noticed (Fig. 1).

*Microscopic.*—No trace of the original ovarian tissues could be identified. The tumour was found to be composed of several intimately intermingled, differentiated tissue elements embedded in loose connective tissue which in several places had a necrotic appearance. These tumour components included hyaline cartilage, bone, and various types of epithelium. Stratified squamous epithelium with well-developed granular and keratin layers and horn pearls usually formed the lining of cystic cavities filled with lamellar material. In other areas plugs or sheets of squamous epithelium were embedded in dense stroma. Throughout the tumour several types of glandular epithelium were conspicuous : high columnar, cuboidal, flat, mucus-secreting (Fig. 3) and ciliated epithelium. As a rule, such cells lined cavities of various shapes and sizes which lumina contained strands of mucus and a few polynuclear leukocytes. In the lining of a single cyst, glandular and squamous epithelium were often seen in continuity. Tubules lined with columnar epithelium and sometimes displaying minimal pseudostratification without the slightest cellular atypia were occasionally noticed. Most tissue sections showed heavily-pigmented squamous and glandular epithelium. Brown pigment granules were also found in stellate stroma cells (melanocytes). In all the fully matured tissues composing the neoplasm, mitotic figures were rare. Scattered throughout the stroma were a few tiny islands of tightly-packed, undifferentiated cells with round to oval-shaped nuclei.

*Diagnosis*

Large, unilateral, solid, differentiated ovarian teratoma occurring in an adult, gravid C3H/N mouse.

*Case* 2

The second tumour was accidentally found in a virgin C3H/N mouse, the uterine cervix of which had been painted twice a week for 18 weeks with a 1 per cent suspension of 3,4-benzopyrene in acetone in an attempt to induce cervico-vaginal malignancy. In the course of this treatment the animal had ingested a total dose of 0·8 mg. of oestriol. A large cervico-vaginal tumour having been palpated through the abdominal wall, the mouse was killed at the age of 23 weeks.

---

EXPLANATION OF PLATES

FIG. 1.—Ovarian teratoma No. 1. Cut surface ; cu : uterine horn ; b : bone spicules ; c : microcysts.

FIG. 2.—Diagram showing topography of tissues in ovarian teratoma No. 2. *Punctated areas :* connective tissue ; *black areas :* squamous epithelium encircling spaces, the lumina of which contain keratinized material. *Lightly striated areas :* glandular epithelium of various types (flat, cuboidal, high columnar, mucus-secreting, and ciliated). *Heavily striated areas :* plugs of fully differentiated squamous epithelium (epidermis) ; sm : smooth muscle fibres ; cm : cross-striated muscle fibres ; mo : mesovarium ; cu : uterine horn ; bo : bursa ovarica ; mk : pigment granules ; tk : sebaceous glands ; bl : vessels ; rk : colonic epithelium ; e : endometrial epithelium ; b : bone spiculae ; k : hyaline cartilage.

FIG. 3.—Ciliated epithelium in ovarian teratoma No. 1 (H. & E. × 830).

FIG. 4.—Cystic cavity (upper right) in teratoma No. 2, partially lined with mucus-secreting epithelium. Growing into the stroma are tubular glands reminiscent of colonic mucosa. Dilated vessels. Lumen of cyst contains strands of mucoid material (H. & E. × 230).

FIG. 5.—Ovarian teratoma No. 2. Glandular space (s) lined with cuboidal epithelium. Intraluminal projection partially covered with epithelium of the same type. Smooth muscle fibres (sm). Heavily pigmented area (p) near and within glandular epithelium (H. & E. 230).

FIG. 6.—Ovarian teratoma No. 2. Stratified keratinized squamous epithelium with well developed granular and keratin layers (top). Cluster of sebaceous glands (bottom). (H. & E. 230).

Thiery

Thiery

*Pathology*

*Gross.*—The uterine corpus, portio, and deep half of the vagina were replaced by a friable tumour mass measuring approximately 10 × 10 × 8 mm. The uterine horns and right ovary were normal. The left ovary was freely movable, ovoid, and slightly enlarged (± 5 × 4 × 4 mm.). Cross-section of this ovarian mass showed a yellowish-grey surface containing several minute cavities. The aortic lymph nodes were grossly enlarged and measured approximately 2 × 4 × 6 mm.

*Microscopic.*—The vaginal epithelium showed slight to severe dysplasia. The cervico-vaginal mass was identified as a differentiated squamous cell carcinoma. In the aortic nodes nothing but inflammatory changes were found. Several sections from the enlarged left ovary contained only normal ovarian tissues, including follicles and corpora lutea ; in other sections a well delineated tumour was found surrounded by an intact ovarian capsule. The tumour had a heterogeneous appearance, more solid parts alternating with small cysts containing mucoid secretion and/or horny material. An idea of its topography is given by the diagram (Fig. 2). The stroma in general consisted of loose connective tissue, hyalinized in several areas. Surrounding spicules of bone and nodules of cartilage, the stroma was usually more fibrous. Numerous dilated and engorged vessels were noticed. Scattered chaotically throughout the stroma were a variety of adult tissues foreign to the ovary and closely resembling their normal counterparts. They included (a) stratified squamous epithelium not unlike epidermis with easily recognizable sebaceous glands (Fig. 6) and pigmented areas, (b) columnar epithelium and stroma cells containing large amounts of brownish pigment granules (Fig. 5), (c) several types of glandular epithelium encircling alveolar and cystic spaces. Most conspicuous were picket-fence cells reminiscent of oestrous endometrial epithelium, high columnar ciliated, and secreting goblet cells. Growing into the underlying stroma were clusters of tubular glands bearing resemblance to colonic mucosa (Fig. 4), (d) islands of hyaline cartilage and pieces of bone, and (e) smooth (Fig. 5) and cross-striated muscle fibres.

*Diagnosis*

Small, unilateral, solid of finely polycystic, fully matured teratoma (teratoma adultum) in an adult virgin C3H/N mouse treated with benzopyrene and oestriol.

### DISCUSSION

Spontaneous, primary, solid ovarian tumours of all types have very rarely been recorded in the mouse (Fischer and Kuehl, 1958 ; Slye *et al.*, 1920 ; Snell, 1956). Ovarian teratomas as a group are extremely rare in this species, and probably no more than 5 cases have been reported in the literature so far (Fawcett, 1950 ; Fekete and Ferrigno, 1952 ; Jackson and Brues, 1941 ; Slye *et al.*, 1920). Only two ovarian teratomas were found in 25,000 autopsied mice of the Slye Stock (Slye *et al.*, 1920) and it is known that the larger part of this colony was composed of strains in which carcinoma was common. In our colony of C3H/N mice, two cases of ovarian teratoma have been observed among some 2000 animals, an incidence of 0·1 per cent.

Our first case closely resembles those reported in the literature, although it should be mentioned that we did not succeed in finding nerve tissues (Fawcett,

1950 ; Fekete and Ferrigno, 1952). Our second teratoma was entirely composed of mature, fully-differentiated tissues, which is a unique feature of such tumours. Moreover, the presence of sebaceous glands in ovarian teratomas has never been recorded in the mouse. On structural grounds this tumour should be considered an example of a solid, histologically benign teratoma. Comparable teratomatous tumours have been reported, however rarely, in the human ovary and in several other sites as well (Peterson, 1956 ; Willis, 1937, 1951), thus stressing the fact that in the mouse as in man, there is an " intermediary " group of teratomas which does not conform to the usual " cystic-benign, solid-malignant " pattern (Matz, 1961).

As one of our tumours originated in a mouse treated with a potent carcinogenic hydrocarbon, it would be unjustifiable to consider this neoplasm as being straightforwardly spontaneous in origin. Although ovarian teratomas have never been experimentally produced in mammals, one feels free to speculate that, at least in this case, teratogenesis could have been accomplished by the chemical metamorphosis of " multipotential " cells. The rare occurrence of ovarian teratomas in painted mice, on the other hand, throws some doubt on the hypothetical role played by carcinogenic compounds in teratogenesis.

SUMMARY

Two primary, solid, histologically benign teratomas of the ovary occurred in two adult members of a colony of C3H/N mice. The first mouse was pregnant, the second had been treated with 3,4-benzopyrene and oestriol.

This report describes the histopathological features of these very rare tumours. It draws attention to the possible teratogenous action on the ovary of the hydrocarbon used.

This work was supported in part by a research grant of the Fonds National de la Recherche Scientifique.

### REFERENCES

FAWCETT, D. W.—(1950) *Cancer Res.*, **10**, 705.
FEKETE, E. AND FERRIGNO, M. A.—(1952) *Ibid.*, **12**, 438.
FISCHER, W. AND KUEHL, I.—(1958) ' Geschwuelste der Laboratoriumsnagetiere '. Dresden (Th. Steinkopff).
JACKSON, E. B. AND BRUES, A. M.—(1941) *Cancer Res.*, **1**, 494.
MATZ, M. H.—(1961) *Obstet. Surv. Baltim.*, **16**, 591.
PETERSON, W. F.—(1956) *Amer. J. Obstet. Gynec.*, **72**, 1094.
SLYE, M., HOLMES, H. F. AND WELLS, H. G.—(1920) *J. Cancer Res.*, **5**, 205.
SNELL, G. D. (Editor)—(1956) ' Biology of the Laboratory Mouse '. New York (Dover Publ., Inc.).
STEWART, H. L., SNELL, K. C., DUNHAM, L. J. AND SCHLYEN, S. M.—(1959) ' Transplantable and transmissible Tumors of Animals.' Washington D.C. (Armed Forces Institute of Pathology Publ.).
WILLIS, R. A.—(1937) *J. Path. Bact.*, **45**, 49.—(1951) ' Teratomas.' Washington D.C. (Armed Forces Institute of Pathology Publ.).